# Editorial: Psychological Aspects and How They Relate to Doping in Sport

**DOI:** 10.3389/fspor.2022.976305

**Published:** 2022-07-22

**Authors:** Kim Nolte, Barend Steyn, Ian David Boardley

**Affiliations:** ^1^Department of Physiology, University of Pretoria, Pretoria, South Africa; ^2^Department of Psychology, University of Pretoria, Pretoria, South Africa; ^3^School of Sport, Exercise, and Rehabilitation Sciences, University of Birmingham, Birmingham, United Kingdom

**Keywords:** doping, psychological factors, sport, values, education

Psychology not only influences an athlete's success in his/her sport, it can also impact the way in which an athlete approaches training and competition and thus can play a role in whether an athlete dopes or not. Researchers in psychology do not yet have a full understanding of why certain athletes dope or have a susceptibility to use performance enhancing drugs (PEDs). Therefore, the main goal of the Research Topic was to gather research on relevant psychological factors and how they relate to doping in athletes.

Culturally relevant values-based education has been cited as a key component for creating a clean sport culture. In a study by Woolway et al. 1,225 athletes from Germany, Greece, Italy, Russia, and the United Kingdom responded to measures assessing their general values, spirit of sport values, and their perceived importance of “clean sport.” There were significant differences between participant nationality and their perceived importance of clean sport, most important general values, and spirit of sport values. Moderate positive correlations were observed between the perceived importance of clean sport and honesty and ethics and respecting the rules of sport. The results highlight the need to better tailor anti-doping interventions to the cultural backgrounds and pre-defining characteristics of an athlete. Constantinou and Aguiyi conducted a separate study that aimed to explore the prevalence, attitudes, and perceptions of the use of both sport and academic PEDs by students at two universities in Johannesburg, South Africa. Five hundred and forty-eight primarily female undergraduate students participated in the study. Six percent indicated current or past use of PEDs. Neuroactive drugs, cannabis and stimulants were the highest used drugs. Academic and cognitive performance were the most common reasons for using PEDs. The participants believed that the use of PEDs was high among their peers. Education and testing programs were interventions most commonly suggested to overcome the use of PEDs in this cohort of students. Lastly, Steyn and Nolte aimed to deepen the understanding of psychological motives of athletes who exhibit tendencies toward cheating in general and the proclivity to use PEDs by means of a historical conceptual analysis of the ego phenomenon. The concept and meanings attached to an ego-involved participant as formulated in the well-established goal orientation theory revealed striking similarities with the ego as elucidated in the historical metaphysical and spiritual writings, 2500 years ago. The analysis of the ego within the major spiritual traditions created a fusion zone between modern psychological theories, approaches, and concepts, and the spiritual traditions of the past. A historical conceptual analysis of the ego phenomenon and the meanings attached to the ego phenomenon can be traced back to spiritual oriental writings. This analysis can deepen the understanding of the psychological motives of athletes who exhibit tendencies toward cheating in general and the proclivity to use prohibited substances.

The findings of these studies indicate (a) that culturally relevant values-based education is important for anti-doping interventions, (b) athletes believe education and testing programs are important to prevent doping, and (c) behavioral analysis of spirituality could possibly enrich several Research Topics in psychology (e.g., self-awareness and therapeutic approaches) and could play a role in deepening the understanding of psychological motives of athletes who dope.

## Author contributions

All authors listed have made a substantial, direct, and intellectual contribution to the work and approved it for publication.

## Conflict of interest

The authors declare that the research was conducted in the absence of any commercial or financial relationships that could be construed as a potential conflict of interest.

## Publisher's note

All claims expressed in this article are solely those of the authors and do not necessarily represent those of their affiliated organizations, or those of the publisher, the editors and the reviewers. Any product that may be evaluated in this article, or claim that may be made by its manufacturer, is not guaranteed or endorsed by the publisher.

